# The Effect of Hexanoyl Glycol Chitosan on the Proliferation of Human Mesenchymal Stem Cells

**DOI:** 10.3390/polym10080839

**Published:** 2018-07-30

**Authors:** Young-Hoon Jeong, Hye Min Oh, Man Ryul Lee, C-Yoon Kim, Chanyang Joo, Soon-Jung Park, Yun-Ho Song, Changhee Kang, Hyung-Min Chung, Sun-Woong Kang, Kang Moo Huh, Sung-Hwan Moon

**Affiliations:** 1 Department of Stem Cell Biology, School of Medicine, Konkuk University, Seoul 05029, Korea; jyh0844@gmail.com (Y.-H.J.); vivavets@gmail.com (C.-Y.K.); parksoonjung@gmail.com (S.-J.P.); Yoontech67@gmail.com (Y.-H.S.); changhee_k@naver.com (C.K.); stemchung@gmail.com (H.-M.C.); 2 Department of Polymer Science and Engineering, Chungnam National University, 99 Daehak-ro, Yuseong-gu, Daejeon 34134, Korea; hyemin0love@naver.com (H.M.O.); Jooyang011@naver.com (C.J.); 3 Soonchunhyang Institute of Medi-bio Science (SIMS), Soon Chun Hyang University, Cheonan 31151, Korea; leeman@sch.ac.kr; 4 Predictive Model Research Center, Korea Institute of Toxicology, 141 Gajeong-ro, Yuseong-gu, Daejeon 34114, Korea; 5 Department of Human and Environmental Toxicology, University of Science and Technology, Daejeon 34114, Korea; 6 Department of Medicine, School of Medicine, Konkuk University, Seoul 05029, Korea

**Keywords:** mesenchymal stem cell, hexanoyl glycol chitosan, basic fibroblast growth factor

## Abstract

Adipose-derived mesenchymal stem cells (AD-MSCs) have been studied as desirable cell sources for regenerative medicine and therapeutic application. However, it has still remained a challenge to obtain enough adequate and healthy cells in large quantities. To overcome this limitation, various biomaterials have been used to promote expansion of MSCs in vitro. Recently, hexanoyl glycol chitosan (HGC) was introduced as a new biomaterial for various biomedical applications, in particular 3D cell culture, because of its biodegradability, biocompatibility, and other promising biofunctional properties. In this study, the effect of HGC on the proliferation of AD-MSCs was examined in vitro*,* and its synergistic effect with basic fibroblast growth factor (bFGF), which has been widely used to promote proliferation of cells, was evaluated. We found that the presence of HGC increased the proliferative capacity of AD-MSCs during long-term culture, even at low concentrations of bFGF. Furthermore, it suppressed the expression of senescence-related genes and improved the mitochondrial functionality. Taken all together, these findings suggest that the HGC demonstrate a potential for sustained growth of AD-MSCs in vitro.

## 1. Introduction

Mesenchymal stem cells (MSCs) have multipotent capacity to differentiate into adipocytes, osteoblasts, and chondrocytes [1,2]. Furthermore, MSC-based therapies are of great interest because of various paracrine effects, modulation of immune responses, and low level of immunogenicity [3,4]. Adipose-derived mesenchymal stem cells (AD-MSCs) are frequently used for various experiments [5,6,7] and clinical applications [8,9,10,11] because (1) they can be easily isolated from tissues and obtained by adipo-suction surgery, (2) they proliferate better than bone-marrow-derived MSCs, (3) they involve the least ethical implications, and (4) more are donated [12,13]. However, even though stem cells can self-renew, their characteristics alter after senescence. For this reason, it is difficult to obtain enough cells for various purposes [14,15]. To overcome these limitations, several cytokines, biomaterials, and manipulation of medium components have been widely used to increase the proliferation of MSCs [16,17,18,19,20,21].

Recently, various biomaterials have been studied to support or control the cellular fate. Among them, chitosan, a deacetylated form of chitin that is the most abundant polymer found in nature after cellulose, has been extensively investigated because of its promising properties of biocompatibility, biodegradability, and other biofunctionalities [22,23]. Most importantly, as a cationic polysaccharide with a porous structure and gel-forming properties, it can be easily chemically modified by adsorption of desired growth factors such as basic fibroblast growth factor (bFGF) and has a high affinity for in vivo macromolecules [24,25]. Furthermore, it mimics the polysaccharide and glycosaminoglycan portion of the extracellular matrix, enabling it to function as a substrate for cell adhesion, migration, and proliferation. Glycol chitosan, a water-soluble chitosan derivative, and its derivatives have been proposed as a suitable material for various pharmaceutical and biomedical applications although very little characterization of their physicochemical and biological properties has been conducted [22]. Recently, Cho et al. developed a series of acyl glycol chitosans (AGCs) with different types of acyl groups and degrees of substitution as a new class of water-soluble chitosan derivatives for three-dimensional (3D) cell-culture systems and other biomedical applications [26,27,28]. Among the AGCs, hexanoyl glycol chitosan (HGC) could provide a facile, convenient, and reproducible method for efficient 3D cell culture and demonstrate better cell proliferation, although there have been no clear systemic studies of its effect on cell proliferation or the functionality related to growth-factor delivery for cell proliferation.

Herein, we demonstrate that HGC combined with bFGF can enable better proliferation of human AD-MSCs and inhibit the expression of senescence-associated genes during the long-term culture. This study suggests that HGC may be a useful biomaterial for producing high-quality and scalable AD-MSCs products. 

## 2. Materials and Methods

### 2.1. Materials

Glycol chitosan (GC, DP ≥ 200, Mn = 86,600 by GPC measurement) was obtained from WAKO Pure Chemical Industries, Ltd. (Osaka, Japan) and hexanoic anhydride (97%) was obtained from Sigma-Aldrich (St. Louis, MO, USA). Spectra/Por membranes were purchased from Spectrum Laboratories, Inc. (Rancho Dominguez, CA, USA). Acetone and methanol were supplied by Samchun Chemical (Pyeongtaek, Korea). All reagents were of analytical grade and used as received.

### 2.2. Synthesis of Hexanoyl Glycol Chitosan (HGC)

HGC was synthesized according to the previously reported procedures and conditions [27]. Briefly, GC (3 g) was dissolved in 750 mL of a mixture of water/methanol (1/1 by volume). A predetermined amount of hexanoic anhydride was then added to the GC solution and kept at room temperature with vigorous stirring for one day. The reaction solution was precipitated in acetone and collected by centrifugation, followed by dialysis (MWCO, 12–14 kDa) against distilled water for purification for two days. The HGC product was obtained by lyophilization in a powder form.

### 2.3. Characterization of HGC

The chemical composition of HGC was characterized by ^1^H NMR spectroscopy using an AVANCE III 600 spectrometer (BRUCKER, Karlsruhe, Germany) operating at 600 MHz. HGC was dissolved in D_2_O at a concentration of 1.0 wt %. The D_2_O peak at *δ* 4.80 was used as a reference peak. ATR-FTIR spectra were also recorded on GC and HGC using a Nicolet iS5 spectrometer (Thermo Fisher Scientific, Waltham, MA, USA) with 16 scans gathered at a resolution of 4 cm^−1^ to confirm the *N*-hexanoylation reaction.

### 2.4. Cell Source and Culture

hAD-MSCs (ATCC, Manassas, VA, USA) were seeded at the density of 4500 cell/cm^2^ on polystyrene tissue-culture dishes and cultured in Minimum Essential Medium *α*-modification (*α*-MEM; Thermo Fisher Scientific) including GlutaMAX, supplemented with 10% fetal bovine serum (FBS; Thermo Fisher Scientific), 1% of penicillin-streptomycin (P/S; Thermo Fisher Scientific), and HGC and/or basic fibroblast growth factor at the indicated concentration. Cells were maintained at 37 °C in a humidified atmosphere containing 5% CO_2_. The culture medium was changed every two days, and cells were cultured to ~90% confluence in the culture medium as above. When −90% confluence was reached, after four days of culture, expanded cells were detached using 0.25% trypsin-EDTA (Thermo Fisher Scientific). Then, collected cells were centrifuged at 1000 rpm, 5 min, and cells were seeded at a density of 4500 cells/cm^2^. Subsequently, every cell was passaged at ~90% confluence until passage 10.

### 2.5. Characterization of Adipose-Derived Mesenchymal Stem Cells (AD-MSCs)

Cells were detached by 0.25% trypsin-EDTA at ~90% confluence of the passage, pellets were resuspended in FACS buffer (PBS solution including 0.5% bovine serum albumin (BSA) and 2 mM EDTA) and filtered using a premoistened a 40-μm cell strainer. Cells were then labelled using each antibody of MSC surface markers according to the manufacturer’s instructions. Types of antibody are as follows. Fluorochrome-conjugated antibodies for CD44-APC, CD73-PE, CD90-APC, and CD105-PE (BD Biosciences, Bedford, MA, USA), along with a negative marker CD31 and CD34 conjugated to APC and PE (BD Biosciences) were used. Also, corresponding IgG controls were prepared equally, and 30,000 labelled cells were acquired and analyzed using Becton Dickinson FACS Calibur (BD Biosciences).

### 2.6. Assessment of Multilineage Differentiation

For the induction of osteoblasts, chondroblasts, and adipocytes, commercially available kits were used (Thermo Fisher Scientific). Cells under differentiation conditions were maintained in 12-well plates. Osteogenesis and chondrogenesis was incubated for 21 days, and adipogenic lineage was induced for 14 days. All experimental procedures were performed according to the manufacturer’s instructions. To evaluate each differentiation process, appropriate staining was performed: Oil Red O to detect intracellular lipid droplets, Von kossa to visualize extracellular mineralized matrix, and Alcian blue to confirm the formation of proteoglycans. Images were analyzed using an inverted microscope (Nikon, Tokyo, Japan).

### 2.7. Cell Proliferation Assay

Cells were counted using a hemocytometer at the beginning and end of each passage. Population doubling time (PDT) was calculated by the formulas PDT = ln2∙*T*/ln(*N_T_*/*N*_0_), where *N_T_* is cell number at the end of a passage, *N*_0_ is the cell number at the seeding density, and *T* is culture time.

### 2.8. Cell Cycle Analysis

AD-MSCs treated with the indicated concentrations of HGC after five passages were detached and fixed in 70% ethanol at 4 °C overnight. After washing with PBS (Thermo Fisher Scientific), cells were resuspended in FxCycle PI/RNase Staining Solution (Thermo Fisher Scientific) and incubated for 30 min at room temperature protected from light. Cells were analyzed by flow cytometry using Becton Dickinson FACS Calibur (BD Biosciences), at least 30,000 cells per sample.

### 2.9. Measurements of Oxygen Consumption Rate (OCR) and Extracellular Acidification Rate (ECAR)

Oxygen consumption rate (OCR) and extracellular acidification rate (ECAR) were analyzed by Seahorse Biosciences kits, following the manufacturer’s instructions. Briefly, AD-MSCs were seeded on XF96 cell-culture microplates (Seahorse Bioscience, North Billerica, MA, USA) and grown to 70% confluence prior to analysis. When cells reached ~70% confluence, the culture medium was replaced with XF Assay medium (Seahorse Bioscience), including 2 mM sodium pyruvate and 2.5 mM glucose for the OCR assay. To quantify the OCR, basal OCR was measured first. Next, a series of changes in OCR was measured when cells were consecutively treated with 1 μM oligomycin, 1 μM, FCCP, and a combination of 1 μM antimycin A and rotenone. The overall oxygen consumption rate (OCR) was analyzed. For measurement of the ECAR, after measuring the basal acidification rate, cells were treated with 10 mM glucose, 2 μM oligomycin, and 100 mM 2-deoxy-glucose (2-DG) sequentially, and serial changes in acidification rate were measured after each addition. For this purpose, changes in culture medium pH were monitored every 20 s and used to calculate the overall extracellular acidification rate (ECAR).

### 2.10. Quantitative Real-Time Polymerase Chain Reaction

Total RNA of each sample was extracted using TRIzol Reagent (Invitrogen, Carlsbad, CA, USA) according to the manufacturer’s instructions. 1 μg of total RNA was transcribed into complementary deoxyribonucleic acid (cDNA) using a High-Capacity cDNA Reverse Transcription Kit (Thermo Fisher Scientific). Real-time PCR was performed by using FastStart Essential DNA Green Master (Roche, Pleasanton, CA, USA) on a LightCycler 96 instrument (Roche). The target genes and associated primers are as follows: *SIRT1* sense 5′-GATAAC CTTCTGTTCGGTGA, *SIRT1* antisense 5′-GAATTGTTCGAGGATCTGTG, *DNMT1* sense 5′-GCT ACCTGGCTAAAGTCAAA, *DNMT1* antisense 5′-ATTCACTTCCCGGTTGTAAG, *DNMT3A* sense 5′-CAGAAGAAGAGAAGAATCCCTAC, *DNMT3A* antisense 5′-CTCATCAATAATCTCCTTGACC, *DNMT3B* sense 5′-GCAGGCAGTAGGAAATTAGA, *DNMT3B* antisense 5′-GGTCTTTGCCGTTGT TATAG, p21 sense 5′-AGAAGAGGCTGGTGGCTATTT, p21 antisense 5′-CCCGCCATTAGCGCATCAC, p53 sense 5′-AGATAGCGATGGTCTGGC, p53 antisense 5′-TTGGGCAGTGCTCGCTTAGT.

### 2.11. Statistical Analysis

All statistical analysis was performed by GraphPad Prism software (La Jolla, CA, USA; Version 5). All data are shown as the mean ± SEM. The statistical significance of the experimental outcomes was calculated using one-way ANOVA. The differences between experimental groups were considered significant when *p* < 0.05.

## 3. Results

### 3.1. Synthesis and Characterization of HGC

**Figure 1:**
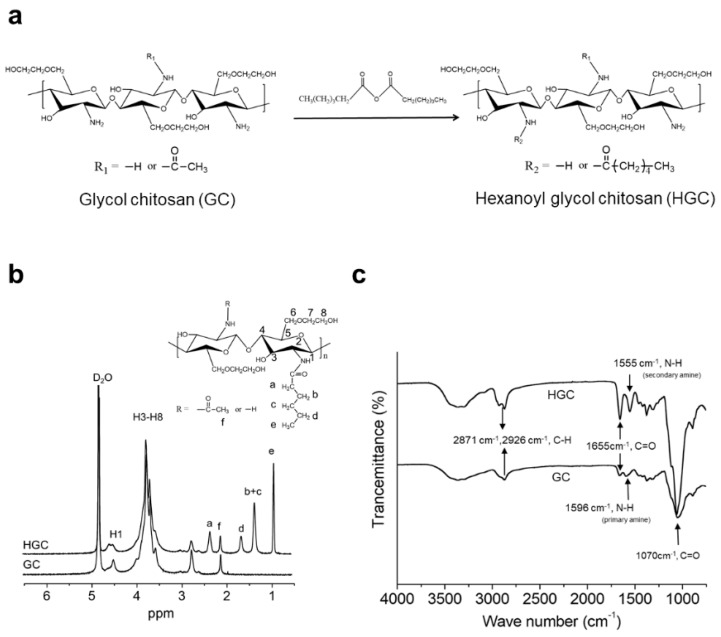
Characterization of hexanoyl glycol chitosan. (**a**) Synthesis procedure of hexanoyl glycol chitosan (HGC)36.9; (**b**) ^1^H NMR spectra and (**c**) FT-IR spectra of HGC36.9 and glycol chitosan (GC).

HGC was synthesized via *N*-hexanoylation of GC using hexanoic anhydride (Figure 1a). The structural composition was characterized by using ^1^H NMR and ATR-FTIR spectrometers. The ^1^H NMR spectra of HGC and GC are shown in Figure 1b. The GC showed the characteristic peaks at *δ* 3.4–4.1 and at *δ* 2.74, corresponding to H3–H8 of the glucopyranosyl ring and H2 (proton) of the glucopyranosyl ring adjacent to the primary amine, respectively. The peak at *δ* 2.02 corresponded to the methyl protons (O=CCH_3_) of the acetyl group. HGC showed new proton peaks from hexanoyl groups at *δ* = 0.89 (CO–CH_2_–CH_2_–CH_2_–CH_2_–CH_3_), *δ* = 1.32 (CO–CH_2_–CH_2_–CH_2_–CH_2_–CH_3_), *δ* = 1.62 (CO–CH_2_–CH_2_–CH_2_–CH_2_–CH_3_) and *δ* = 2.31 (CO–CH_2_–CH_2_–CH_2_–CH_2_–CH_3_). The degree of substitution (DS) of HGC was calculated to be 36.9% by comparing the integrated signal area at glucopyranosyl ring protons and hexanoyl group protons. The DS value was the similar to the previously reported one (DS = 36.5). As shown in Figure 1c, the ATR-FTIR spectra of GC and HGC showed the absorbance at 2871 cm^−1^ and 2926 cm^−1^ attributed to the C–H stretching of methyl and methylene groups, respectively. Although the absorption peak at 1596 cm^−1^ corresponds to the primary amine bending vibration of GC, the absorbance at 1555 cm^−1^ and 1655 cm^−1^ corresponds to the secondary amine and carbonyl stretching vibration of the amide groups of the HGC. The results from ^1^H NMR and ATR-FTIR measurements showed that the *N*-hexanoylation reaction occurred successfully. 

### 3.2. Characterization of Human Adipose-Derived Mesenchymal Stem Cells

**Figure 2:**
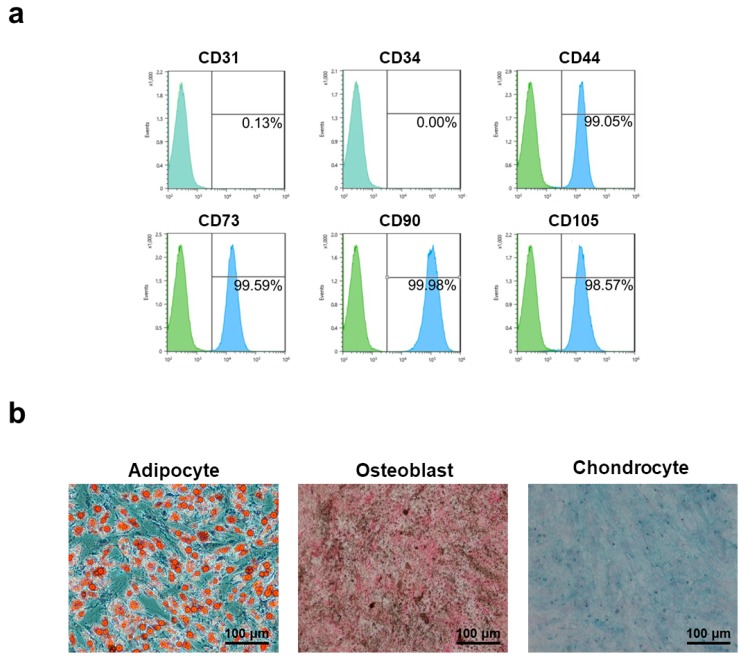
Characterization of adipose-derived mesenchymal stem cells (AD-MSCs) used for experiments. (**a**) Positive markers for CD44, CD73, CD90, and CD105, negative markers for CD31 and CD34; (**b**) Staining for multilineage differentiation of AD-MSCs.

AD-MSCs used in this study were characterized by the marker expression and lineage differentiation to verify properties of AD-MSCs. Cell population displayed a general MSC antigen profile that exhibited CD44, CD73, CD90, and CD105 expression, as well as a lack of CD31 and CD34 for the negative markers (Figure 2a). These results indicate that the cells expressed all the proper markers of MSCs and did not express any other markers of differentiation. To further investigate the multipotent properties of AD-MSCs, the cells were differentiated into osteoblasts, adipocytes, and chondrocytes as demonstrated by von kossa staining, oil red O staining, and Alcian blue staining, respectively (Figure 2b). Taken all together, these results show that the acquired MSCs had normal marker expression and differentiation potential.

### 3.3. The Effect of HGC Following Long-Term Passaging of AD-MSCs

**Figure 3:**
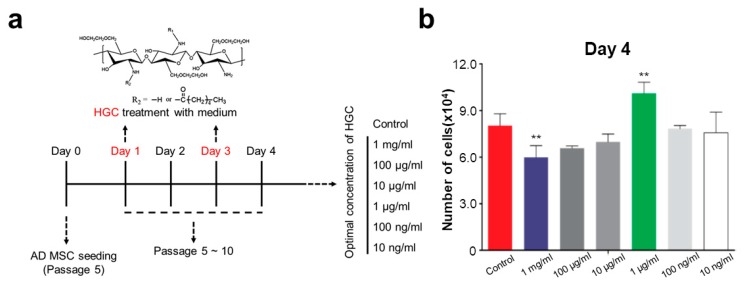

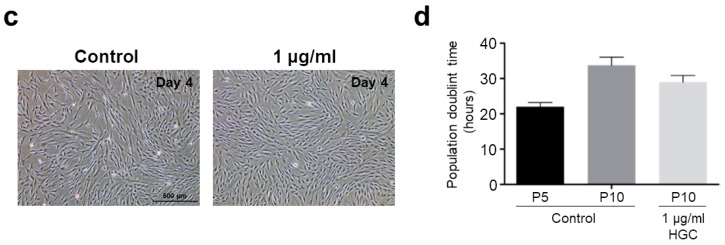
Investigation of optimal hexanoyl glycol chitosan (HGC) concentration. (**a**) experimental scheme; (**b**) cell number of AD-MSCs at day four (passage 5); ** *p* < 0.01 relative to the control; (**c**) cell morphology and density of the control and 1 μg/mL of HGC at day four (passage 5); (**d**) Comparison of population doubling time at passage 5 (the control) and the control and 1 μg/mL of HGC group at passage 10.

The effect of HGC on the proliferation rate and the doubling time of AD-MSCs following passages was observed as a function of the polymer concentration. To investigate the proliferation rate of AD-MSCs after long-term culture, we treated HGC during AD-MSCs culture. Various concentrations of HGC ranging from 10 ng/mL to 1 mg/mL were treated at passage 5 (Figure 3a). We observed that cells proliferated more rapidly than the control when HGC was treated at 1 μg/mL for four days (Figure 3b,c). Furthermore, population doubling time (PDT) of the HGC-treated group decreased in comparison to the control after five passages (Figure 3d), which demonstrates that the HGC treatment with an optimal concentration of 1 μg/mL may enhance the proliferation and thus decrease the population doubling time.

### 3.4. Combination Effect of HGC with Basic Fibroblast Growth Factor for Proliferation of AD-MSCs

**Figure 4:**
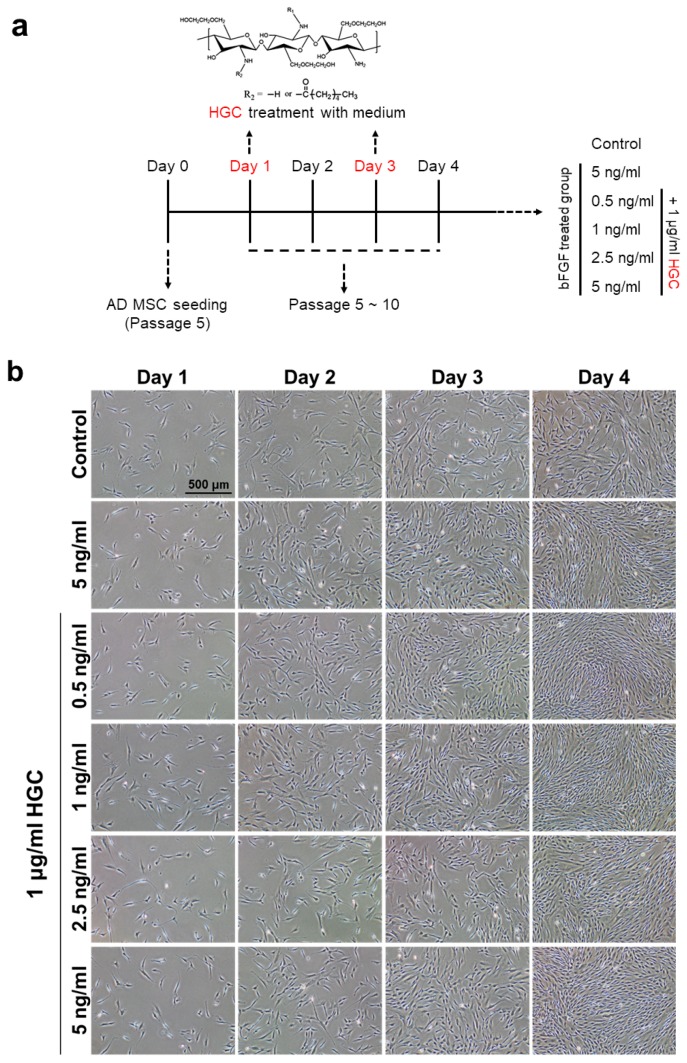

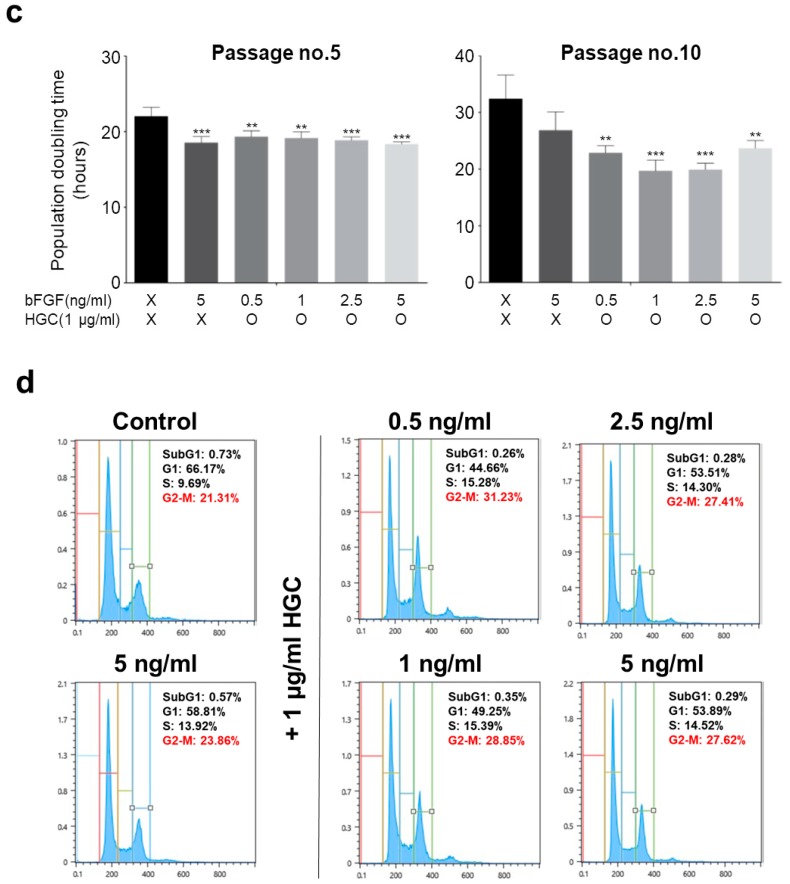
Comparison of proliferative capacity between HGC combined with basic fibroblast growth factor (bFGF) groups and non-treated groups. (**a**) Experimental scheme following various concentration of bFGF treatment with 1 μg/mL of HGC; (**b**) Daily incubated cell morphology picture of AD-MSCs at passage 5; (**c**) Population doubling time at passages 5 and 10; and (**d**) cell cycle analysis. ** *p* < 0.01, and *** *p* < 0.001 relative to the control.

Because basic fibroblast growth factor (bFGF) has been widely used to accelerate the growth of mesenchymal stem cells, we investigated the effect of HGC combined with bFGF. The concentration of bFGF used varied by sequential dilution (Figure 4a). All bFGF-treated groups showed that the number of cells increased significantly more than that of the control after four days of culture at passage 5 regardless of the HGC treatment. It is, however, notable that there was no significant difference of cell density between the HGC/bFGF-treated groups with different concentrations of bFGF and the bFGF-only group at passage 5 (Figure 4b). Moreover, although the bFGF-treated groups showed similar PDT at passage 5, the PDT of HGC/bFGF-treated groups decreased much more than that of the control and the bFGF-only groups after five passages (Figure 4c). Interestingly, compared with a high concentration of bFGF, even when bFGF concentration was low, we could obtain scalable cell numbers when they were cultured with HGC. To further investigate the proliferation of AD-MSCs following HGC treatment, we performed cell-cycle assays. Consistently, cell-cycle analysis revealed that the G2-M phase was significantly more up-regulated in HGC-treated groups than in non-treated groups (Figure 4d). Especially, the G2-M phase significantly increased in 0.5 ng/mL of bFGF with HGC. In addition, cells treated with 1 ug/mL of HGC-only showed that the HGC-only group had a slightly higher cell population in the G2-M phase in comparison to the control but lower than the bFGF-only group (Figure S1). Altogether, our data demonstrate that the presence of HGC with the minimal concentration of bFGF may efficiently accelerate the proliferation of AD-MSCs.

### 3.5. Oxidative Consumption Rate and Extracellular Acidification Rate Analysis

**Figure 5:**
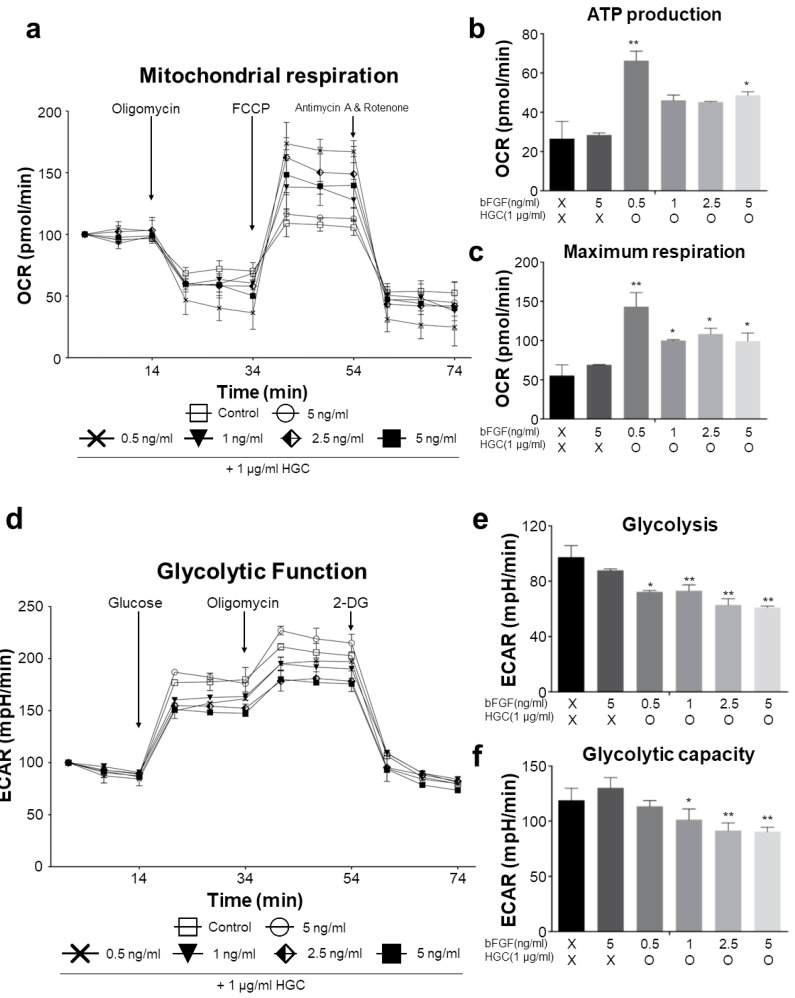
The analysis of metabolic functionality of AD-MSCs at passage 10. (**a**) The measurement of oxygen consumption rate following oligomycin, FCCP, antimycin A, and rotenone treatment; (**b**) ATP production; (**c**) maximum respiration; (**d**) The measurement of extracellular glycolytic acidification following glucose, oligomycin, and 2-DG treatment. Glycolytic function displayed an increased extracellular acidification rate in control and 5 ng/mL bFGF-only treated group; (**e**) glycolysis and (**f**) glycolytic capacity. * *p* < 0.05 and ** *p* < 0.01 relative to the control. FCCP: carbonyl cyanide-4 (trifluoromethoxy) phenylhydrazone, 2-DG: 2-deoxy-glucose.

To measure the metabolic change of AD-MSCs following long-term HGC treatment, we analyzed the oxidative consumption rate (OCR) and extracellular acidification rate (ECAR) by using Extracellular Flux Analyzer at passage 10. In OCR analysis (Figure 5a), after oligomycin treatment, the oxygen consumption rate of the HGC-treated groups, especially with 0.5 ng/mL of bFGF, was more inhibited than that of non-treated groups, indicating higher ATP production in mitochondria (Figure 5b). Next, in the presence of FCCP, a potent uncoupler of oxidative phosphorylation in mitochondria, 0.5 ng/mL of bFGF with HGC had much more OCR data than did the non-treated groups (Figure 5c), demonstrating that the maximal respiratory function of mitochondria was increased. On the other hand, from ECAR analysis (Figure 5d), in the presence of glucose, glycolytic acidification was lower for HGC-treated groups than for non-treated groups (Figure 5e). Moreover, glycolytic capacity also decreased in HGC-treated groups, indicating that the HGC non-treated groups generated energy using glycolysis rather than mitochondrial respiration (Figure 5f). Altogether, we suggest that bFGF is more effective with the presence of HGC for the long-term culture of AD-MSCs because it also increases mitochondrial function even at relatively low concentrations of bFGF.

### 3.6. Quantitative Analysis of Aging-Related Gene Expression

**Figure 6:**
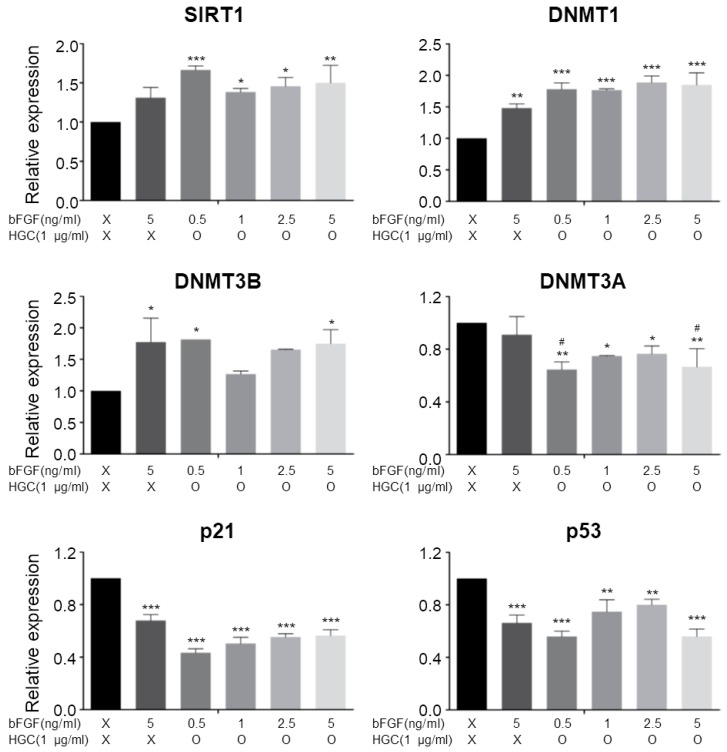
Quantitative real-time polymerase chain reaction (RT-PCR) analysis for senescence markers. * *p* < 0.05, ** *p* < 0.01, and *** *p* < 0.001 relative to the control. ^#^ *p* < 0.05 relative to bFGF-only group.

In order to verify whether stimulation of AD-MSCs growth by the HGC/bFGF combination treatment is correlated with gene expression of cellular senescence, we assessed SIRT1, DNMT1, DNMT3B, DNMT3A, p21, and p53 mRNA levels by using quantitative real-time PCR (Figure 6). The expression of Sirtuin 1 (SIRT1) was significantly up-regulated in cells treated with HGC, especially for the 0.5 ng/mL of bFGF group. Analysis of the expression of DNA methyltransferases (DNMTs) revealed that DNMT1 and DNMT3B extensively increased more for all bFGF-treated groups than for the control. In contrast, DNMT3A, which participates in the new methylation, was down-regulated in the HGC-treated groups. Additionally, both p21 and p53 were down-regulated in all bFGF-treated groups more than in the control. Especially, 0.5 ng/mL of bFGF with HGC displayed the most significant down-regulation. Altogether, these data indicate that the HGC/bFGF combination treatment may contribute to regulating the cellular senescence of AD-MSCs.

### 3.7. Immunophenotype and Multi-Differentiation Capacity of AD-MSCs after Long-Term Culture

**Figure 7:**
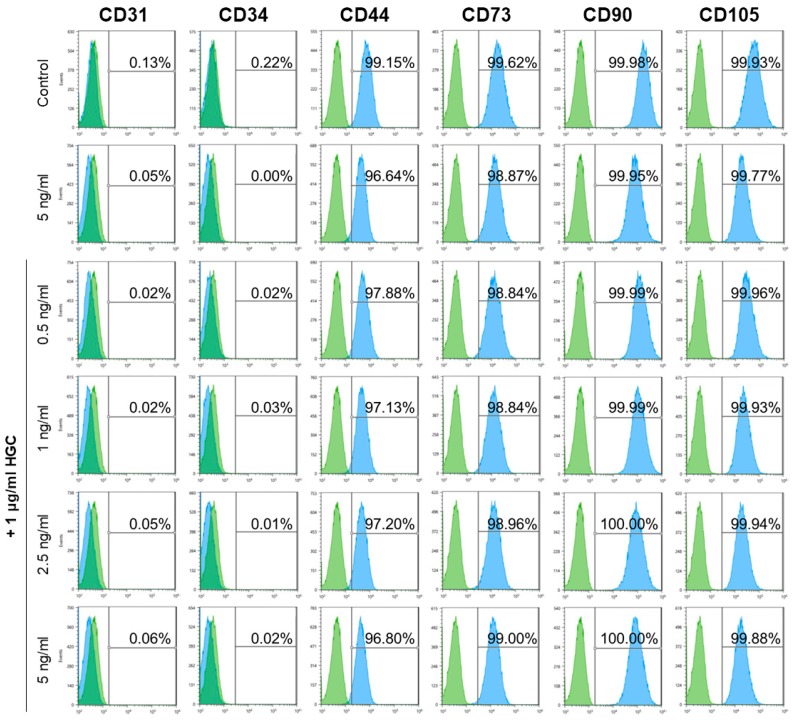
Characterization of AD-MSCs phenotypes by fluorescence-activated cell sorting (FACS) after long-term culture (passage 10).

In order to confirm whether the characteristics of AD-MSCs were maintained after five passages with HGC treatment in vitro, we analyzed cell-surface marker expression by flow cytometry for each group at passage 10. All groups expressed CD44, CD73, CD90, and CD105 with an average of >95% for positive markers and lack of CD31 and CD34 for negative markers (Figure 7), which demonstrates the stability of the CD profile of AD-MSC after long-term culture with HGC. Furthermore, we investigated the multilineage differentiation capacity of each group at passage 10 (data not shown). We found that all groups were well differentiated into osteoblasts, adipocytes, and chondrocyte as confirmed by von kossa, Oil Red O, and Alcian blue staining, respectively.

## 4. Discussion

Ex vivo expansion of MSC is needed to acquire enough cells because of the limitation of cell sources in the body. However, cellular senescence occurs in various cell types including MSCs in vitro [29], the proliferative capacity is suppressed, and thus the doubling time is increased. For these reasons, various attempts have been made to obtain enough cells by using cytokines, biomaterials, and manipulation of medium components [16,17,18,19,20,21,26,30]. In this study, we demonstrated that the HGC/bFGF combination treatment can increase the proliferation capacity of AD-MSCs and inhibit the expression of senescence associated genes during long-term culture.

As shown in Figure 4c, both HGC/bFGF-treated groups and the bFGF-only group decreased in PDT compared with the control, but the specific efficacy of HGC was not confirmed, indicating that HGC treatment for four days did not affect the proliferation of AD-MSCs. However, after five passages, HGC-treated groups showed decreased doubling time compared with that of non-treated groups, which implies that HGC may contribute to proliferative capacity and cell senescence during long-term culture. In addition, a relatively abundant G2-M phase in the HGC- treated groups consistently support our data about increased proliferative capacity (Figure 4d) [31,32]. 

For the metabolic analysis, comparatively high ATP production and maximum respiration by the HGC-treated groups may be attributed to rapid cell proliferation [33]. Also, because biological energy was mainly generated by mitochondrial respiration, glycolytic capacity relatively decreased. These findings mean that HGC may increase the mitochondrial functionality during long-term culture. We also analyzed MSCs surface-marker expression to verify whether cells were differentiated into other lineages. As shown in Figure 7, consistent phenotypes with non-treated groups after five passages support our hypothesis. In addition, the maintenance of the capacity of HGC-treated groups to differentiate at passage 10 may demonstrate that HGC both facilitates the proliferation capacity of AD-MSCs and preserves their differentiation capacity after long-term culture, because it has been reported that differentiation potential was suppressed after long-term culture [34].

SIRT1 regulates telomere aging and protects cells from DNA damage [35]. Thus, the up-regulation of SIRT1 attenuates the biological aging process, resulting in increased proliferation of AD-MSCs. Furthermore, because DNA methylation is closely involved in replicative senescence, we analyzed the gene expression of DNA methyltransferases (DNMTs). The increase of DNMT1 and DNMT3B, which are substantially down-regulated during replicative senescence, revealed that HGC may suppress cellular senescence [36]. In addition, a decrease of DNMT3a, which is involved in the new methylation and plays an opposite role to DNMT3B, consistently supports our hypothesis [37,38]. Moreover, down-regulation of p21 and p53 suppresses cellular senescence and is a reason for the increased proliferation of the HGC-treated groups. These data suggest that HGC could regulate the expression of senescence-related gene through cellular affinity.

In this study, we confirmed that the HGC/bFGF combination treatment can increase the proliferation capacity of AD-MSCs. Moreover, HGC influenced expression of senescence-associated genes and improved mitochondrial functionality. Interestingly, we found that HGC can effectively increase the proliferation of AD-MSCs even at a relatively low level of bFGF. This may be because HGC is better delivered to the cells due to its well-documented interaction with the glycosaminoglycans groups of the extracellular matrix [39,40,41], thus affecting cell proliferation. This study suggests that HGC may prove to be very useful as a biomaterial to produce high-quality and scalable AD-MSCs products.

## Supplementary Materials

The following are available online at http://www.mdpi.com/2073-4360/10/8/839/s1, Figure S1: Additional cell cycle analysis of 1 ug/mL hexanoyl glycol chitosan (HGC) only groups at passage 10.
