# Supplementary material for: The Effect of Hexanoyl Glycol Chitosan on the Proliferation of Human Mesenchymal Stem Cells
Source: Polymers (Basel). 2018 Jul 30;10(8):839. doi: 10.3390/polym10080839 (PMC6404012; doi:10.3390/polym10080839)
Supplement: Supplementary file 1 [file polymers-10-00839-s001.pdf]

polymers

MDPI

Supplementary Material

The Effect of Hexanoyl Glycol Chitosan on the Proliferation of Human Mesenchymal Stem Cells

Young-Hoon Jeong [1,†], Hye Min Oh [2,†], Man Ryul Lee [3,†], C-Yoon Kim [1], Chanyang Joo [2], Soon-Jung Park [1], Yun-Ho Song [1], Changhee Kang [1], Hyung-Min Chung [1], Sun-Woong Kang [4,5,*,‡], Kang Moo Huh [2,*,‡], and Sung-Hwan Moon [6,*,‡]

[1] Department of Stem Cell Biology, School of Medicine, Konkuk University, Seoul 05029, Korea; vivavets@gmail.com (C.-Y.K.); parksoonjung@gmail.com (S.-J.P.); Yoontech67@gmail.com (Y.-H.S.); changhee_k@naver.com (C.K.); stemchung@gmail.com (H.-M.C.)
[2] Department of Polymer Science and Engineering, Chungnam National University, 99 Daehak-ro, Yuseong-gu, Daejeon 34134, Korea; Jooyang011@naver.com (C.J.); khuh@cnu.ac.kr (K.M.H.)
[3] Soonchunhyang Institute of Medi-bio Science (SIMS), College of Medicine, Soon Chun Hyang University, Cheonan 31151, Korea
[4] Predictive Model Research Center, Korea Institute of Toxicology, 141 Gajeong-ro, Yuseong-gu, Daejeon 34114, Korea; swkang@kitox.re.kr
[5] Department of Human and Environmental Toxicology, University of Science and Technology, Daejeon 34114, Korea
[6] Department of Medicine, School of Medicine, Konkuk University, Seoul 05029, Korea; sunghwanmoon@kku.ac.kr
* Correspondence: swkang@kitox.re.kr (S.-W.K.); khuh@cnu.ac.kr (K.M.H.); sunghwanmoon@kku.ac.kr (S.-H.M.); Tel.: +82-42-610-8209 (S.-W.K.); Tel.: +82-42-821-8910 (K.M.H.); Tel.: +82-2-2049-6028 (S.-H.M.)
† These authors contributed equally to this work as first author.
‡ These authors contributed equally to this work as corresponding authors.

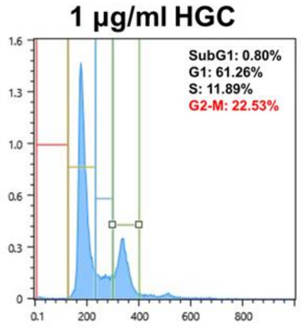

Figure S1. Additional cell cycle analysis of 1 ug/mL hexanoyl glycol chitosan (HGC) only groups at passage 10.
